# Risk Stratification and Patient Trajectories in CAPS: The Important Role of the Autoinflammatory Disease Damage Index

**DOI:** 10.3390/jcm15218232

**Published:** 2026-10-22

**Authors:** Özlem Satirer, Marie Bogenschütz, Susanne M. Benseler, Jasmin B. Kuemmerle-Deschner

**Affiliations:** 1 Division of Pediatric Rheumatology and Autoinflammation Reference Center Tuebingen (arcT), Department of Pediatrics, University Children’s Hospital Tuebingen, Hoppe-Seyler-Strasse 1, 72076 Tuebingen, Germany; marie.bogenschuetz@med.uni-tuebingen.de (M.B.);; 2 European Reference Network for Rare Immunodeficiency, Autoinflammatory and Autoimmune Diseases, ERN-RITA Coordinating Office, 3584 CX Utrecht, The Netherlands; 3 Rheumatology, Department of Pediatrics, Alberta Children’s Hospital (ACH), ACH Research Institute, Cumming School of Medicine, University of Calgary, Calgary, AB T3B 6A8, Canada; 4 Children’s Health Ireland, D12 N512 Dublin, Ireland

**Keywords:** cryopyrin-associated periodic syndrome, autoinflammatory disease damage index, risk stratification, treat-to-target, ADDI

## Abstract

**Objective**: Persistent inflammation in cryopyrin-associated periodic syndromes (CAPS) causes cumulative organ damage, quantified by the Autoinflammatory Disease Damage Index (ADDI). We evaluated the ADDI for risk stratification and assessed risk trajectories under treat-to-target (T2T) management. **Methods**: This retrospective analysis of prospectively collected single-centre registry data included consecutive paediatric patients diagnosed with CAPS between 2007 and 2024 with ≥24 months of follow-up. The ADDI was scored at the initial damage assessment, 6 months after the first assessment at our centre (baseline), and at the last visit, and patients were assigned to predefined mild, moderate and severe risk strata. **Results**: Of 71 patients (56% female; median follow-up 7 years), 23 (32%) had damage at the initial damage assessment, most frequently auditory (24%), developmental (14%) and ocular (11%). ADDI scores were higher in patients with pathogenic/likely pathogenic *NLRP3* variants (median 2 vs. 0; *p* < 0.001) and increased across phenotypes (FCAS 0, MWS 0, CINCA/NOMID 9; *p* < 0.001). Risk strata remained unchanged in 69 patients (97%). Two children progressed from mild to moderate risk due to progression of sensorineural hearing loss; both were non-adherent to anti-IL-1 therapy, and neither showed increased disease activity at the last visit. **Conclusions**: In paediatric CAPS, early cumulative damage was common and associated with phenotype and *NLRP3* variant pathogenicity. Under T2T management, ADDI risk strata remained largely stable. Although based on few patients, the damage progression captured by the ADDI, but not reflected by conventional disease activity measures at the time of assessment, supports a complementary role of the ADDI in longitudinal outcome monitoring.

## 1. Introduction

Autoinflammatory diseases are a heterogeneous group of rare disorders characterised by recurrent or persistent systemic inflammation, driven predominantly by dysregulation of innate immune pathways [1]. In monogenic autoinflammatory diseases, genetic defects affecting inflammasome activation and cytokine signalling can lead to excessive production of pro-inflammatory mediators, particularly interleukin-1β, interleukin-18, and tumour necrosis factor-α [1]. Cryopyrin-associated periodic syndrome (CAPS) is a prototypical monogenic autoinflammatory disease caused by gain-of-function variants in *NLRP3*, which drive unregulated interleukin-1β secretion and produce a clinical continuum spanning Familial Cold Autoinflammatory Syndrome, Muckle–Wells syndrome and neonatal-onset multisystem inflammatory disease [2]. Persistent inflammation may result in cumulative organ damage, including sensorineural hearing loss, central nervous system sequelae, musculoskeletal restriction or deformity, growth failure, pubertal delay, renal involvement, and AA amyloidosis [2,3]. These complications are major determinants of long-term morbidity, the latter highlighting the need to assess cumulative damage in addition to inflammatory activity.

The Autoinflammatory Disease Damage Index (ADDI) was developed through an international expert consensus initiative as the first instrument for the structured quantification of cumulative organ damage in hereditary autoinflammatory diseases [4]. It comprises 18 items grouped into eight organ domains. Damage is defined as a persistent or irreversible structural or functional change present for at least six months and not attributable to ongoing inflammatory activity. Notably, according to the original scoring principle, once an item has fulfilled the definition of damage and has been scored, it continues to contribute to the cumulative ADDI score even if the abnormality later improves or resolves [4]. ADDI reliability and validity were subsequently supported by an in silico validation study using paper-based clinical cases assessed by international experts [5].

Although the ADDI is established as a measure of damage assessment, its role in longitudinal outcome monitoring remains insufficiently defined. Damage accrual may define risk strata that can identify patients at risk of adverse outcomes. It may capture clinically meaningful trajectories while providing treat-to-target (T2T) management, a role that has not been evaluated in paediatric patients. Paediatric CAPS represents a particularly suitable setting to address this gap, given the early disease onset, lifelong disease course, and susceptibility to cumulative damage accrual.

Therefore, the aims of the present study were to describe a single-centre cohort of consecutive paediatric CAPS patients, to evaluate ADDI as an instrument for risk stratification, and to assess the trajectories of risk strata while receiving T2T disease management.

## 2. Materials and Methods

This was a retrospective analysis of prospectively collected longitudinal clinical data from a single-centre registry. The study included consecutive paediatric patients diagnosed with CAPS between 01/2007 and 12/2024 at the Autoinflammation Reference Centre Tuebingen (arcT), a national referral centre for autoinflammatory diseases, who met the CAPS diagnostic criteria. Paediatric CAPS was defined as symptom onset before the age of 18 years. Patients had to have a minimum follow-up of 24 months, allowing serial assessments at the 6-, 12-, and 24-month and last follow-up visits. CAPS patients with systemic conditions that independently affect any of the ADDI domains were excluded. Data were prospectively captured at each visit in the institutional Arthritis and Rheumatism Database and Information System (ARDIS). Ethics approval was obtained from the Medical Faculty Ethics Committee of Eberhard Karls University Tuebingen (Project Number: 531/2016BO2).

**Diagnosis and definition of time points.** CAPS was diagnosed according to the CAPS diagnostic criteria, which require elevated inflammatory markers (CRP/SAA) plus at least two of six CAPS-typical signs or symptoms (urticaria-like rash, cold-/stress-triggered episodes, sensorineural hearing loss, musculoskeletal symptoms, chronic aseptic meningitis and skeletal abnormalities) and do not require a confirmed NLRP3 variant [6]. Accordingly, in patients carrying NLRP3 variants of uncertain significance (VUSs) or without an identifiable variant, the diagnosis was established clinically by experienced paediatric rheumatologists at the reference centre based on these criteria. Elevated inflammatory markers were accepted irrespective of the centre at which they were measured. In addition, fulfilment of the Eurofever/PRINTO classification criteria was assessed [7]. Baseline was defined as the first assessment at our centre, which in most patients coincided with the first diagnosis of CAPS. The initial damage assessment was performed 6 months after baseline, in line with the ADDI requirement that damage must be present for at least 6 months.

## 3. Data and Assessments

Patient- and disease-related data included gender, age at symptom onset, and age at diagnosis. The CAPS phenotype, including mild (Familial Cold Autoinflammatory Syndrome, FCAS), moderate (Muckle–Wells Syndrome, MWS), or severe (Chronic Infantile Neurologic Cutaneous and Articular Syndrome, CINCA), was recorded. *NLRP3* variants and their classification according to the American College of Medical Genetics and Genomics/Association for Molecular Pathology (ACMG/AMP) criteria were documented [8]. The type, dose, and start and stop dates of all treatments; dose adjustments; switches between agents; and treatment adherence were documented at each visit. Treatments included colchicine and IL-1 inhibitors. Time to treatment was defined as the interval between the onset of first symptoms and the initiation of therapy. Data were captured serially.

**Genetic testing.** Genetic testing was performed using a next-generation sequencing (NGS) panel of autoinflammatory disease genes including *NLRP3* and/or genome sequencing, including analysis for low-level somatic (mosaic) *NLRP3* variants. Variants were classified according to the ACMG/AMP criteria [8]. The family history of CAPS-typical symptoms in first- and second-degree relatives was recorded.

## 4. Monitoring Instruments and Definitions

Disease activity was captured using internationally accepted standard measures used in CAPS clinical trials and practice, including the Physician Global Assessment (PGA), the Patient/Parent Global Assessment (PPGA), and a patient-reported fatigue score. All three measures were assessed using a 10 cm visual analogue scale (VAS), where 0 indicated no disease activity (or no fatigue) and 10 represented maximal disease activity (or maximal fatigue). Physician and Patient/Parent Global Assessments form the core of established CAPS response and remission definitions [9] and of consensus treat-to-target protocols [10]. Laboratory parameters comprised inflammatory markers, including serum amyloid A (SAA) and C-reactive protein (CRP). CRP is reported in mg/dL and SAA in mg/L throughout. Complete remission was defined as PGA ≤ 2, with CRP ≤ 0.5 mg/dL and/or SAA ≤ 10 mg/L. Partial remission was PGA > 2 and ≤5, with CRP > 0.5 mg/dL but ≤3 mg/dL and/or SAA > 10 mg/L but ≤30 mg/L. Non-remission was defined as PGA > 5, with CRP > 3 mg/dL and/or SAA > 30 mg/L [9].

**Treat-to-target management.** All patients were managed according to an institutional T2T strategy consistent with the German PRO-KIND consensus protocols and the EULAR/ACR points to consider [3,10]. The treatment target was complete remission as defined above. Patients were reviewed every 3–6 months depending on disease activity; if the target was not achieved, treatment was escalated by switching from colchicine to an IL-1 inhibitor and/or by increasing the dose or shortening the dosing interval of the IL-1 inhibitor. Colchicine was used as initial therapy in patients with mild disease activity carrying low-penetrance *NLRP3* variants, with escalation to IL-1 blockade if the target was not reached; patients with mild, infrequent symptoms were monitored without maintenance treatment. Treatment adherence was assessed at each visit by patient/parent interview and by review of patient-held CAPS treatment diaries, in which the date and batch number of each administered dose were recorded.

## 5. Damage Assessment

The Autoinflammatory Disease Damage Index (ADDI), a validated, disease-specific instrument developed for use across autoinflammatory diseases, was applied to assess cumulative organ damage [4,5]. Damage was defined according to the ADDI as a persistent or irreversible change in structure or function present for ≥6 months and not attributable to ongoing disease activity. As per ADDI guidance, damage could result from previous disease activity, treatment-related complications, or comorbidities; for all items persisting for ≥6 months, scores must be carried forward even if they subsequently resolved as per ADDI guidance [4,5]. The ADDI comprises eight systemic and organ-specific domains, each with a predefined maximum score. The total score can range from 0 to 27. The reproductive domain (maximum 2) included sub/infertility (2 points) and amenorrhoea (1 point). The renal/amyloidosis domain (maximum 6) comprised limited or extensive amyloidosis (2–3 points), proteinuria (1 point), and moderate or severe renal insufficiency (2–3 points). The developmental domain (maximum 3) included growth failure (2 points) and pubertal delay (1 point). The serosal domain (maximum 1) consisted of serosal scarring (1 point). The neurological domain (maximum 6) comprised developmental delay (2 points), cognitive impairment (3 points), elevated intracranial pressure (2 points), and central nervous system involvement (3 points). The auditory domain (maximum 2) assessed moderate or severe sensorineural hearing loss (1–2 points). The ocular domain (maximum 3) included ophthalmologist-confirmed ocular damage graded according to severity (1–3 points). The musculoskeletal domain (maximum 4) comprised joint restriction (2 points), bone deformity (2 points), osteoporosis with fracture and/or vertebral collapse (1 point), and non-inflammatory musculoskeletal pain affecting daily activities (1 point) [4,5].

ADDI items were assessed within routine care using standardised examinations: age-appropriate audiological testing (pure-tone audiometry; behavioural/play audiometry or otoacoustic emissions in young children) at least annually; ophthalmological examination including slit-lamp examination and fundoscopy at least annually; urinalysis for proteinuria at every visit; height, weight and pubertal staging at every visit; and neurological examination and, where indicated, cranial MRI, lumbar puncture and neurocognitive testing.

## 6. Risk Stratification and Patient Trajectories

Using the ADDI as a risk-stratification instrument, patients were assigned to one of three mutually exclusive risk strata applied in a hierarchical manner. Severe risk was defined as ≥2 points in at least one of the eight ADDI domains, irrespective of the total score. Moderate risk reflected an ADDI score of ≥1 and mild risk a score of <1. The strata were defined a priori by the study team on the basis of clinical expertise and the ADDI item weighting, rather than derived empirically from the cohort data. A score of ≥2 points within a single domain corresponds to items weighted by the ADDI as severe organ damage (e.g., severe sensorineural hearing loss, growth failure, developmental delay, cognitive impairment, CNS involvement, amyloidosis or joint restriction), which are likely to be associated with relevant functional impairment irrespective of the number of affected domains; such patients were therefore classified as ‘severe risk’ regardless of their total score. Patients classified as moderate risk had one or more lower-weighted (1-point) items only (e.g., moderate hearing loss, proteinuria or pubertal delay). ADDI was scored at the initial damage assessment (6 months after baseline) and at the last study visit; transitions between strata were recorded. All patients received treat-to-target (T2T) management. Patients were assigned to two groups based on time from onset of symptoms to initiation of therapy of either <5 years or ≥5 years. The 5-year cut-off was chosen pragmatically a priori as a clinically meaningful threshold for substantially delayed treatment; to avoid loss of information through dichotomisation, time to treatment was additionally analysed as a continuous variable.

## 7. Outcomes

The primary outcome was the ADDI total score at initial damage assessment, 6 months after baseline. Secondary outcomes included (1) ADDI domain score at initial damage assessment and (2) risk strata as defined by ADDI score at initial damage assessment and last follow-up.

## 8. Analysis

Categorical variables were summarised as frequencies and continuous variables as means ± SD or medians (min–max), as appropriate. Normality was assessed using the Shapiro–Wilk and Kolmogorov–Smirnov tests. Within-group comparisons over time were performed using paired *t*-tests or Wilcoxon signed-rank tests. Independent groups were compared using the Mann–Whitney U test (two groups) or the Kruskal–Wallis test (more than two groups), correlations were assessed using Spearman’s rank correlation coefficient, and categorical associations were assessed using the chi-square or Fisher’s exact test, as appropriate. Variant-specific analyses were considered descriptive. Sensitivity analyses were performed excluding carriers of the low-penetrance variant Q703K. A two-sided *p*-value < 0.05 was considered statistically significant. Analyses were performed using SPSS Statistics version 28.0.1.1(IBM Corp., Armonk, NY, USA).

## 9. Results

A total of 71 paediatric CAPS patients were included in the study, comprising 31 males (44%) and 40 females (56%). Two children were excluded due to systemic conditions such as severe Foetal Alcohol Syndrome (FASD). The median age at CAPS disease onset was 2 years (min–max: 0–15) and the median age at diagnosis was 5 years (min–max: 0–23; one patient with symptom onset at 10 years was diagnosed at 23 years). The median time to treatment was 2 years (min–max: 0–14) and the median follow-up duration was 7 years (min–max: 2–17). Genetic testing revealed that 20 patients (28%) carried pathogenic/likely pathogenic *NLRP3* variants, 41 (58%) harboured *NLRP3* VUSs and 10 (14%) had no identifiable *NLRP3* variant. A positive family history of CAPS-typical symptoms was reported in 16 of 20 patients (80%) with pathogenic/likely pathogenic variants, 17 of 41 (41%) with VUSs and 2 of 10 (20%) without an identifiable variant. All 71 patients fulfilled the CAPS diagnostic criteria, and 35 (49%) additionally fulfilled the Eurofever/PRINTO classification criteria; elevated inflammatory markers were documented during at least one flare in all patients. The clinical features supporting the diagnosis are shown by genetic subgroup in Supplementary Table S1. Phenotypical classification revealed that 77% of patients (*n* = 55) had a moderate CAPS phenotype, while 11 patients (15%) exhibited a mild and 5 (7%) a severe phenotype. At baseline, the Patient/Parent Global Assessment (PPGA) and Physician Global Assessment (PGA) scores were in the high range with a median of 6 (min–max: 0–9) for PPGA and 6 (min–max: 0–9) for PGA. The median fatigue score was 8 (min–max: 1–10). The median baseline CRP level was 0.47 mg/dL (min–max: 0.01–17.1). Regarding initial treatment, 37 patients (52%) received IL-1 inhibitors, 18 (25%) colchicine and 16 (23%) no maintenance treatment (Table 1). All colchicine-treated patients carried the low-penetrance VUS Q703K; of the untreated patients, 9 had no identifiable *NLRP3* variant.

**Disease activity under T2T management.** At baseline, 7 patients (10%) fulfilled the definition of complete remission, 25 (35%) of partial remission and 39 (55%) of non-remission. At the last visit, 63 patients (89%) were in complete remission, 6 (8%) in partial remission and 2 (3%) in non-remission; thus, not all patients achieved the predefined treatment target.

## 10. Distribution and Organ-Specific Patterns of Initial Damage

Among 71 children with CAPS, 23 (32%) had damage in at least one ADDI domain at initial damage assessment, whereas 48 (68%) had none. The most frequently affected domains were auditory, developmental and ocular. Auditory damage was recorded in 17 patients (24%), developmental damage including growth failure and pubertal delay in 10 (14%), and ocular damage in 8 (11%). Neurological damage was present in 6 patients (8%) and musculoskeletal damage in 5 (7%). By contrast, renal/amyloidosis damage was rare (3 patients, 4%); reproductive and serosal damage were each observed in a single patient (1%) (Table 2).

## 11. Variables Associated with ADDI Total Scores

**Figure 1:**
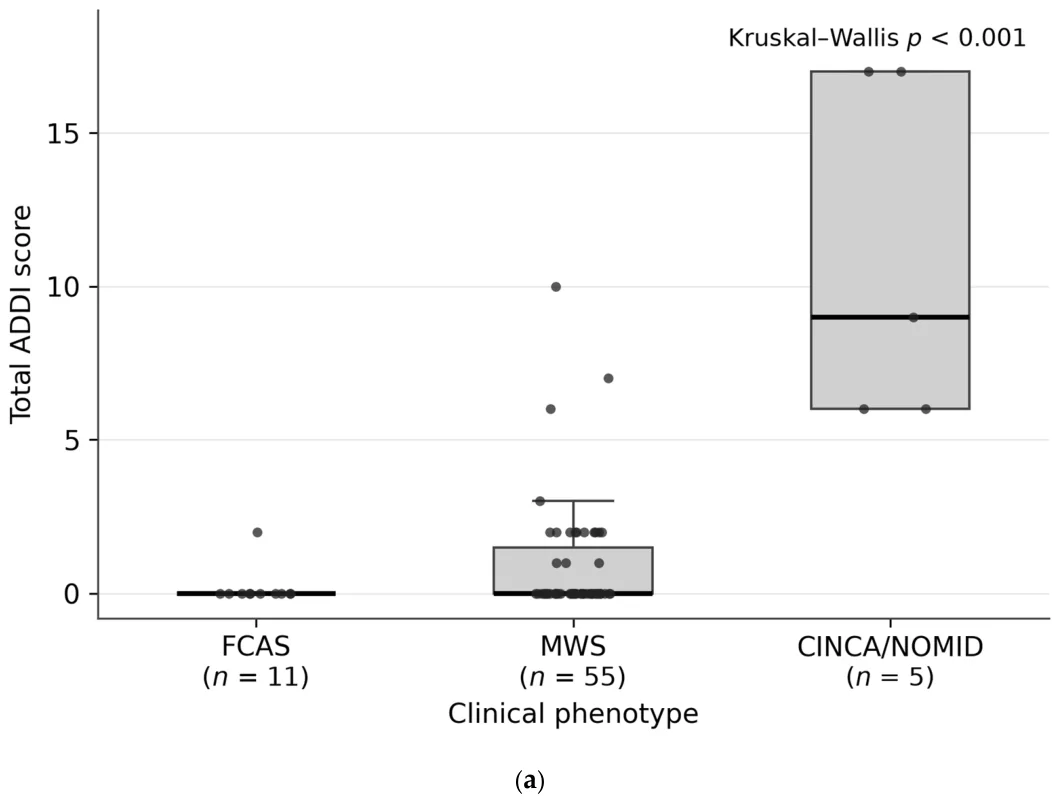

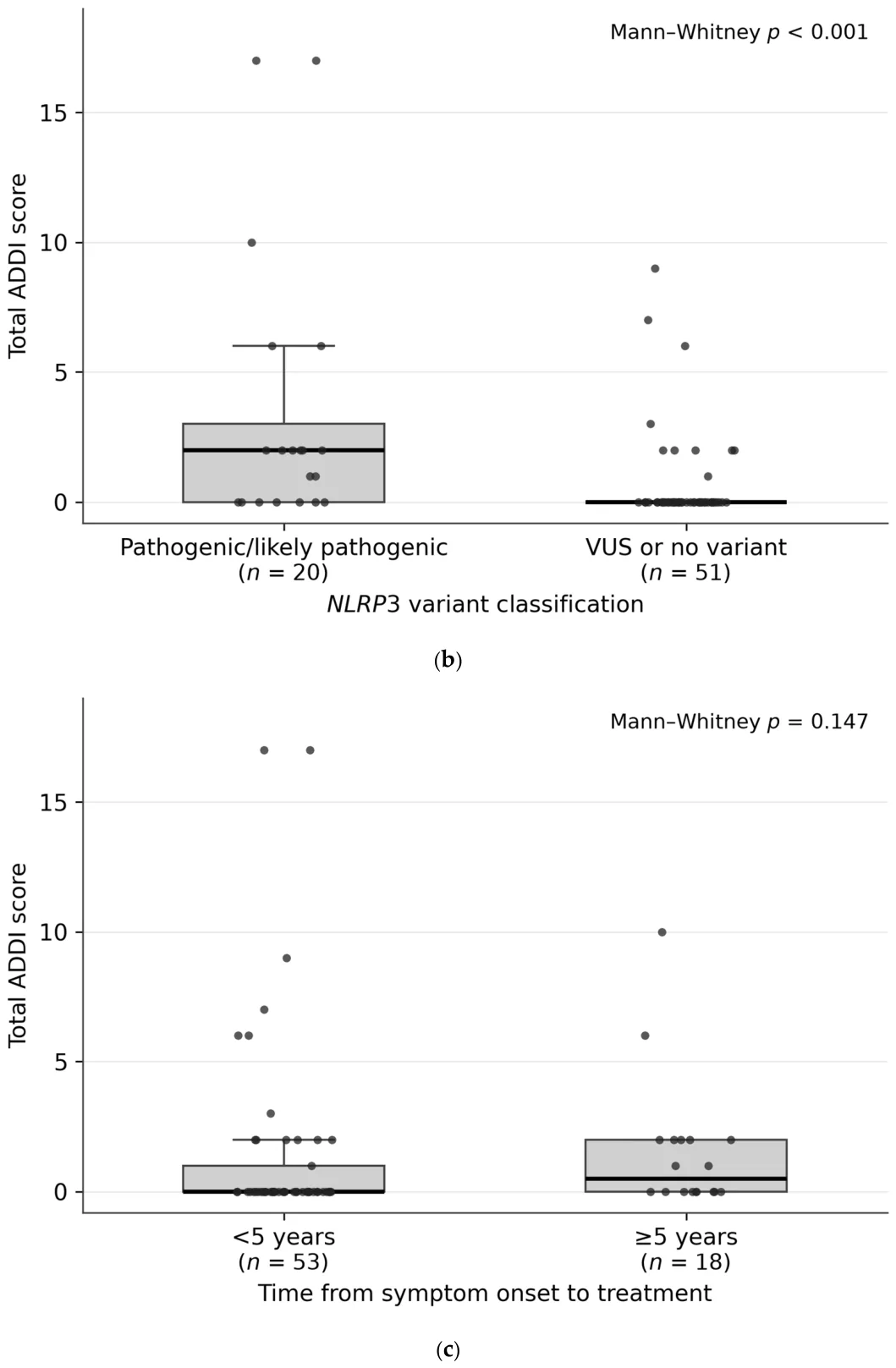
Clinical, genetic and treatment-related variables associated with ADDI total scores in children with CAPS. (**a**) Impact of clinical CAPS phenotypes. **Legend:** Phenotypes are grouped as mild (FCAS), moderate (MWS), and severe (CINCA). A significant increase in ADDI scores was observed with advancing clinical severity (*p* < 0.001, Kruskal–Wallis test). Boxes indicate the median and interquartile range, whiskers 1.5 × IQR; dots represent individual patients. (**b**) Impact of pathogenicity of *NLRP3* variant. **Legend:** Distribution of ADDI total scores in paediatric CAPS patients by *NLRP3* variant pathogenicity. Patients carrying pathogenic/likely pathogenic *NLRP3* variants (*n* = 20) had significantly higher ADDI scores than those with VUSs or no identifiable variant (*n* = 51) (median 2 [min–max 0–17] vs. 0 [min–max 0–9]; Mann–Whitney U test, *p* < 0.001), indicating greater irreversible organ damage. (**c**) Impact of time to treatment. **Legend**: Boxplots display the median, interquartile range, 1.5 × IQR whiskers, and individual patient values. Median total ADDI scores were higher in patients treated ≥5 years after symptom onset than in those treated earlier (0.5 vs. 0), although this difference was not statistically significant (*p* = 0.147). The observed trend may have been influenced by differences in phenotype distribution between the treatment-delay groups: FCAS, 82% vs. 18%; MWS, 73% vs. 27%; and CINCA/NOMID, 80% vs. 20% (<5 vs. ≥5 years).

**Clinical phenotype:** Total scores differed significantly across the clinical CAPS phenotypes—Familial Cold Autoinflammatory Syndrome (FCAS), Muckle–Wells Syndrome (MWS), and Chronic Infantile Neurologic Cutaneous and Articular (CINCA/NOMID) syndrome—at the initial damage assessment (*p* < 0.001). The median total score (min–max) was 0 (0–2) for FCAS (*n* = 11), 0 (0–10) for MWS (*n* = 55), and 9 (6–17) for CINCA/NOMID (*n* = 5) (Figure 1a).

**Pathogenicity of *NLRP3* variant:** ADDI total scores differed significantly between patients with pathogenic/likely pathogenic *NLRP3* variants (*n* = 20) and those with VUSs or no identifiable variant (*n* = 51) (*p* < 0.001), with a median ADDI score of 2 (min–max 0–17) in the pathogenic/likely pathogenic group compared with 0 (min–max 0–9) (Figure 1b). The specific *NLRP3* variants differed in their initial ADDI scores; descriptively, the highest scores were observed in the single patients carrying the M664T and H358R variants (17 each), with intermediate scores for N654S (*n* = 1; VUS), Y570C (*n* = 1) and T348M (*n* = 2; median) (9, 6 and 5, respectively). As most variant groups comprised only one or a few patients, these findings are descriptive and do not allow conclusions on variant-specific effects (Supplementary Table S3).

**Time to treatment:** In CAPS patients treated ≥5 years after symptom onset, the median total ADDI score tended to be higher than in those treated earlier (0.5 vs. 0); however, the difference was not statistically significant (*p* = 0.147) (Figure 1c). Analysed as a continuous variable, time to treatment was not correlated with the initial ADDI total score (Spearman’s ρ = 0.16, *p* = 0.18). This association may have been confounded by the unequal distribution of disease phenotypes between the groups, with the more severe phenotypes being recognised and treated earlier. Specifically, 73% of MWS and 80% of CINCA/NOMID patients received treatment within 5 years of symptom onset.

## 12. Risk Strata and Patient Risk Trajectories

**Figure 2:**
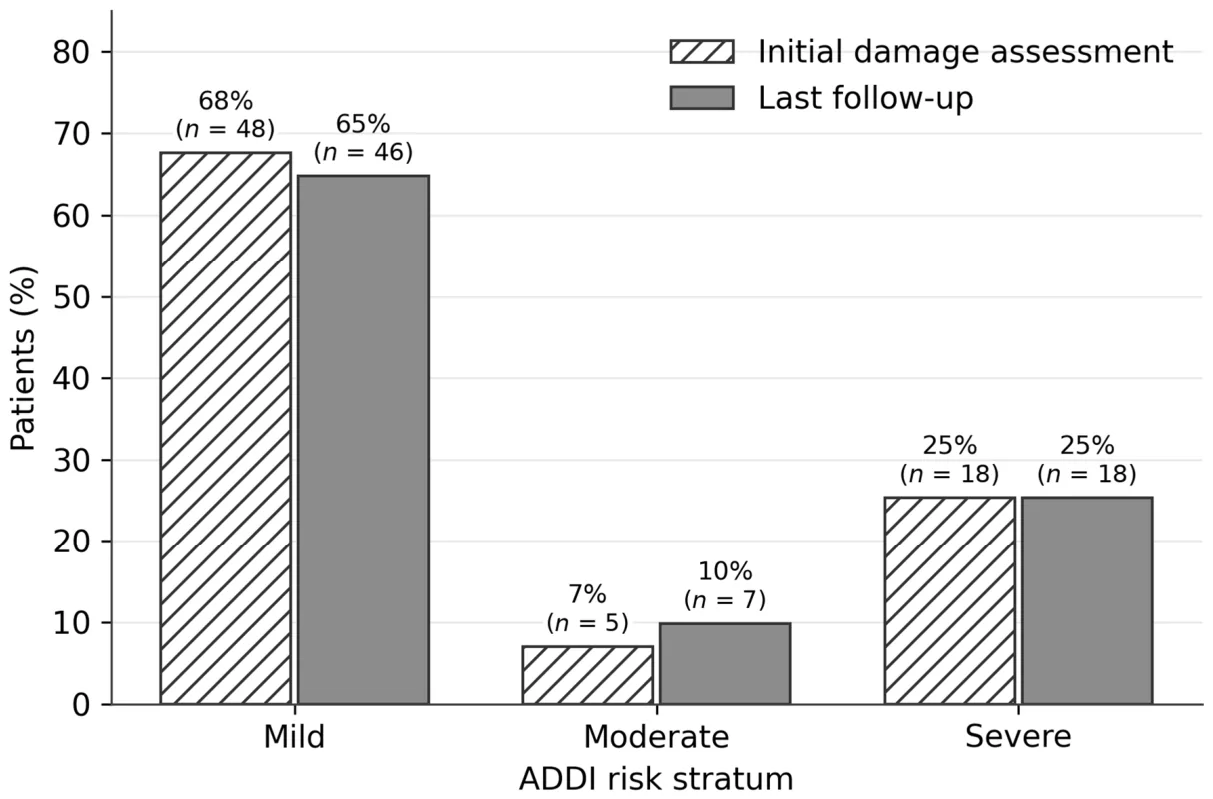
ADDI risk strata at the initial damage assessment and last visit in paediatric CAPS patients. **Legend:** Distribution of ADDI risk strata at the initial damage assessment and last follow-up (*n* = 71). Bars represent the proportion of patients (%) in each risk category; hatched bars indicate the initial damage assessment and grey bars the last follow-up. The proportion of patients in the severe risk category remained unchanged (**25%**), indicating no overall shift in ADDI risk stratification during follow-up. **ADDI**, Autoinflammatory Disease Damage Index.

At the initial damage assessment, 48 of 71 patients (68%) were classified as being at mild risk, whereas 5 (7%) and 18 (25%) were classified as being at moderate and severe risk, respectively. Total ADDI scores ranged from 1 to 2 in the moderate and from 2 to 17 in the severe stratum (Supplementary Table S2). At the last follow-up, 46 patients (65%) were classified as being at mild risk, 7 (10%) at moderate risk, and 18 (25%) at severe risk (Figure 2). Overall, the distribution of ADDI risk strata remained largely stable between the initial damage assessment and the last follow-up under a treat-to-target (T2T) management strategy. At an individual patient level, 69 of 71 patients (97%) remained in the same risk category. However, two patients (3%) progressed from the mild- to the moderate-risk trajectory. Notably, this progression was not accompanied by changes in disease activity, including inflammatory laboratory markers. Further evaluation revealed that both patients had been non-adherent to their prescribed canakinumab treatment, which they had received for approximately 7 years (in one patient, treatment had been initiated before referral to our centre). One patient had an MWS phenotype and a pathogenic *NLRP3* variant (T348M), the other an FCAS phenotype and a VUS (V198M); at the last visit, both had a PGA of 0 and normal CRP and SAA. In both patients, the new damage consisted of progression of sensorineural hearing loss (auditory domain, 1 point). Non-adherence consisted of irregular administration and temporary self-discontinuation of canakinumab.

**Subgroup with pathogenic/likely pathogenic *NLRP3* variants.** Among the 20 patients carrying pathogenic/likely pathogenic variants (median ADDI 2, min–max 0–17), 7 were classified as mild, 3 as moderate and 10 as severe risk at the initial damage assessment; 19 (95%) remained in the same stratum at the last follow-up. One of the two patients with stratum progression (T348M) belonged to this subgroup.

**Sensitivity analysis.** After exclusion of carriers of the low-penetrance variant Q703K (*n* = 35), 20 of the remaining 36 patients were classified as mild, 3 as moderate and 13 as severe risk at the initial damage assessment; 34 (94%) remained in their initial stratum, and ADDI scores continued to differ across phenotypes (*p* < 0.001).

## 13. Discussion

To our knowledge, this is the first longitudinal study to evaluate damage accrual in a paediatric CAPS cohort using the Autoinflammatory Disease Damage Index (ADDI). Damage was present in approximately one-third of patients at the initial damage assessment, most frequently affecting the auditory, developmental and ocular domains, whereas renal/amyloidosis damage remained rare. ADDI scores were significantly associated with *NLRP3* variant pathogenicity and clinical phenotype severity, which is consistent with the construct validity of the index in this population. Under sustained treat-to-target (T2T) management, risk strata remained largely stable over time; the two patients who progressed to a higher stratum were both non-adherent to therapy, and their new damage was not accompanied by changes in conventional disease activity measures. These findings underscore the value of systematic damage assessment alongside disease activity monitoring in the long-term management of children with autoinflammatory disease.

ADDI scores were closely associated with the underlying genotype and clinical phenotype. Damage burden increased across the CAPS spectrum, from FCAS through MWS to CINCA/NOMID, supporting the importance of a genotype–phenotype–damage continuum. This observation is consistent with the clinical continuum of CAPS described previously, in which specific gain-of-function *NLRP3* variants have been mapped to progressively more severe phenotypes [11]. At the molecular level, such variants drive excessive inflammasome activation and sustained interleukin-1β release, providing a biological basis for the greater inflammatory burden observed in the more severe phenotypes [12]. Accordingly, the clinical severity gradient across CAPS phenotypes has been linked to increasing cumulative organ involvement [2]. Although the association between treatment delay and ADDI scores did not reach statistical significance, there was a trend toward greater damage accrual among patients with longer symptom-to-treatment intervals, supporting the importance of early diagnosis and timely initiation therapy. In this study, the association may have been confounded by the unequal distribution of disease phenotypes between the treatment groups, potentially masking a true effect of treatment delay on damage accrual.

Only 28% of patients carried pathogenic/likely pathogenic *NLRP3* variants, whereas the majority carried VUSs or had no identifiable variant. This reflects the referral population of a national reference centre, in which most VUSs were the low-penetrance *NLRP3* variants Q703K (*n* = 35) and V198M (*n* = 5), which are frequently associated with milder, predominantly MWS-like phenotypes [13]. Q703K carriers often present with an atypical, fever-predominant phenotype that may overlap with PFAPA-like disease; nevertheless, 7 of the 35 Q703K carriers had damage at the initial damage assessment, 5 of whom were classified as severe risk, and the results were consistent when Q703K carriers were excluded. As the CAPS diagnostic criteria do not require a confirmed *NLRP3* variant [6], these patients were diagnosed clinically; in variant-negative patients, somatic *NLRP3* mosaicism was excluded by testing for low-level somatic variants. The lower ADDI scores in patients without pathogenic/likely pathogenic variants are consistent with this milder spectrum, and the subgroup analysis restricted to carriers of pathogenic/likely pathogenic variants yielded consistent results, with 19 of 20 patients remaining in their initial stratum. Colchicine is not standard therapy in CAPS, for which IL-1 blockade is recommended [3]. Within our T2T approach, colchicine was used as initial therapy only in patients with mild disease activity carrying the low-penetrance variant Q703K (*n* = 18), with escalation to IL-1 inhibition if the treatment target was not achieved.

The ADDI appears to be a valuable tool for routine risk stratification and longitudinal monitoring of damage accrual in CAPS. Within this T2T-managed cohort, ADDI risk strata remained largely stable over time; however, in the absence of a control group and given the cumulative nature of the ADDI, this observation does not allow conclusions on a protective effect of T2T management. Notably, the only patients who progressed to a higher risk stratum were non-adherent to therapy, and this deterioration was detected by the ADDI despite stable disease activity measures and inflammatory markers. As this observation is based on two patients only, it should be interpreted with caution. Because low perceived disease activity may itself contribute to non-adherence, these cases illustrate the value of damage assessment independent of symptom burden. This pattern aligns with, and adds a longitudinal dimension to, damage indices developed in other chronic paediatric rheumatic diseases. In juvenile idiopathic arthritis, for instance, the Juvenile Arthritis Damage Index has similarly been shown to capture cumulative organ-level sequelae that are not reliably predicted by disease-activity measures alone [14]. This is reinforcing the broader principle that damage and activity represent related but non-interchangeable dimensions of disease burden. Consistent with this, the ADDI was explicitly developed to be used alongside validated measures of disease activity, on the premise that accrued organ damage constitutes a distinct outcome dimension that is not captured by activity indices alone [4]. Collectively, these findings support the integration of structured longitudinal damage assessment alongside disease activity monitoring as a key component of T2T-guided care.

This study has several limitations. Its design as a retrospective analysis of prospectively collected single-centre data and the small number of patients with a severe CAPS phenotype limit generalisability and constrain the power of subgroup analyses. The risk-stratum cut-offs applied here were defined a priori based on expert opinion and the ADDI item weighting and have not been externally validated; they should therefore be regarded as hypothesis-generating. In addition, the cumulative and non-reversible nature of the ADDI precludes the detection of clinical improvement, so the observed stability of risk strata reflects, in part, the design of the index itself. Finally, the modest number of stratum transitions limits firm conclusions regarding the determinants of damage progression. Disease activity was assessed using global VAS scores and inflammatory markers, which may be susceptible to recall bias, particularly in patients with mild, episodic symptoms; a more detailed instrument such as the AIDAI [15] was not routinely captured during the study period. Most patients carried VUSs or no identifiable *NLRP3* variant and were diagnosed clinically, and variant-specific analyses were limited by small numbers. Dichotomisation of time to treatment may have reduced statistical power. Moreover, treatment availability and clinical practice changed over the long inclusion period (2007–2024), including the approval of canakinumab for CAPS in the European Union in 2009, which may have influenced treatment choices and outcomes. Despite these limitations, the findings support the value of incorporating the ADDI into longitudinal T2T monitoring in paediatric CAPS and provide a rationale for validation in larger, multicentre cohorts.

## 14. Conclusions

In paediatric CAPS, early cumulative damage was common and associated with genotype and phenotype. With T2T management, ADDI-based risk strata remained largely stable. In the two patients whose risk stratum changed, the ADDI captured new damage that was not reflected in conventional disease activity measures. Although these observations are based on few patients, they support a complementary role of the ADDI for risk stratification and longitudinal outcome monitoring.

## Figures and Tables

**Table 1:** Baseline demographic, clinical, genetic, and treatment characteristics of 71 children with cryopyrin-associated periodic syndrome (CAPS).

Characteristic	Value
Total (*n*)	71
Gender, F/M	40/31
Age at Symptom Onset (years), median (min–max):	2 (0–15)
Age at Diagnosis (years), median (min–max):	5 (0–23)
Time to Treatment (years), median (min–max):	2 (0–14)
Follow-up Duration (years), median (min–max):	7 (2–17)
***NLRP3*** **variant classification, *n* (%)** Pathogenic/likely pathogenic (P/LP) Variant of Uncertain Significance (VUS) None	20 (28%) 41 (58%) 10 (14%)
**CAPS Phenotype, *n* (%)** Mild (FCAS) Moderate (MWS) Severe (CINCA/NOMID)	11 (15%) 55 (77%) 5 (7%)
Fatigue Score at Baseline, median (min–max)	8 (1–10)
PPGA at Baseline (0–10 cm VAS), median (min–max)	6 (0–9)
PGA at Baseline (0–10 cm VAS), median (min–max)	6 (0–9)
CRP at Baseline (mg/dL), median (min–max)	0.47 (0.01–17.1)
**Initial therapy, *n* (%)** Colchicine IL-1 inhibitors None	18 (25%) 37 (52%) 16 (23%)

Abbreviations: CINCA, chronic infantile neurologic cutaneous and articular syndrome; CRP, C-reactive protein; F, female; FCAS, familial cold autoinflammatory syndrome; IL-1, interleukin-1; M, male; MWS, Muckle–Wells syndrome; NOMID, neonatal-onset multisystem inflammatory disease; PGA, Physician Global Assessment; PPGA, Patient/Parent Global Assessment; VAS, visual analogue scale.

**Table 2:** Distribution and organ-specific patterns of initial damage assessed with the Autoinflammatory Disease Damage Index (ADDI) in paediatric patients with CAPS.

Category	*n* (%)	Score (Median, min–max)
**ADDI total score**		
No damage (ADDI = 0)	48 (68%)	0 (0–0)
Any damage present (ADDI ≥ 1)	23 (32%)	2 (1–17)
**ADDI domains**		
Auditory (sensorineural hearing loss)	17 (24%)	0 (0–2)
Developmental (growth failure, pubertal delay)	10 (14%)	0 (0–3)
Ocular (ophthalmologic damage)	8 (11%)	0 (0–2)
Neurological (CNS involvement, cognitive/developmental impairment, ICP)	6 (8%)	0 (0–6)
Musculoskeletal (joint/bone involvement, osteoporosis, pain)	5 (7%)	0 (0–4)
Renal (amyloidosis, proteinuria, renal insufficiency)	3 (4%)	0 (0–1)
Reproductive (infertility, amenorrhoea)	1 (1%)	0 (0–1)
Serosal (serosal scarring)	1 (1%)	0 (0–1)

**Legend:** Damage was assessed with the ADDI at the initial damage assessment (6 months after baseline) and is reported for the cohort overall and for each of the eight organ domains. For the overall index, patients were dichotomised into *no damage* (ADDI = 0) and *damage present* (ADDI ≥ 1); *n (%)* denotes the number and proportion of patients in each category, and the score is given as median (min–max). For the individual domains, *n (%)* denotes the number and proportion of patients with any damage in that domain, and the domain sub-score is given as median (min–max) across the whole cohort (a median of 0 reflects the absence of damage in the majority of patients). The ADDI assigns weighted points to 18 items across eight organ systems, yielding domain maxima of 1 (serosal), 2 (reproductive; auditory), 3 (developmental; ocular), 4 (musculoskeletal) and 6 (renal/amyloidosis; neurological), and a theoretical maximum total score of 27. At the initial damage assessment, 23 of 71 patients (32%) already showed irreversible organ damage (median ADDI 2, min–max 1–17), most frequently affecting the auditory domain (sensorineural hearing loss, 24%), followed by the developmental (14%) and ocular (11%) domains. Item definitions follow the original ADDI publication (ter Haar et al., 2017) [4]. ADDI, Autoinflammatory Disease Damage Index; CAPS, cryopyrin-associated periodic syndrome; CNS, central nervous system; ICP, intracranial pressure.

## Data Availability

Source data are available from the corresponding author upon reasonable request.

## Supplementary Materials

The following supporting information can be downloaded at: https://www.mdpi.com/article/10.3390/jcm15218232/s1. Table S1. Clinical features supporting the diagnosis of CAPS by NLRP3 variant classification. Table S2A. Distribution of ADDI total scores at the initial damage assessment and corresponding risk strata (n = 71). Table S2B. Individual ADDI domain and total scores of patients with damage at the initial damage assessment or with a change in risk stratum during follow-up. Table S3. NLRP3 variants, number of carriers, clinical phenotype and ADDI total score at the initial damage assessment.
