# Supplementary material for: Risk Stratification and Patient Trajectories in CAPS: The Important Role of the Autoinflammatory Disease Damage Index
Source: J Clin Med. 2026 Oct 22;15(21):8232. doi: 10.3390/jcm15218232 (PMC13763947; doi:10.3390/jcm15218232)
Supplement: Supplementary file 1 [file jcm-15-08232-s001.zip › jcm-4511116-supplementary.pdf]

Supplementary Materials

Risk Stratification and Patient Trajectories in CAPS: The Important Role of the Autoinflammatory Disease Damage Index

Table S1. Clinical features supporting the diagnosis of CAPS by *NLRP3* variant classification

Feature, n (%)	P/LP (n = 20)	VUS (n = 41)	No identifiable variant (n = 10)	Total (n = 71)
Elevated CRP/SAA during flares	20 (100%)	41 (100%)	10 (100%)	71 (100%)
Urticaria-like rash	19 (95%)	16 (39%)	9 (90%)	44 (62%)
Cold-/stress-triggered episodes	17 (85%)	35 (85%)	10 (100%)	62 (87%)
Arthralgia/arthritis/myalgia	17 (85%)	40 (98%)	7 (70%)	64 (90%)
Sensorineural hearing loss	7 (35%)	5 (12%)	1 (10%)	13 (18%)
Chronic aseptic meningitis	3 (15%)	2 (5%)	0 (0%)	5 (7%)
Conjunctivitis	16 (80%)	10 (24%)	5 (50%)	31 (44%)
Fever episodes	10 (50%)	38 (93%)	10 (100%)	58 (82%)
Fatigue	19 (95%)	36 (88%)	8 (80%)	63 (89%)
Positive family history	16 (80%)	17 (41%)	2 (20%)	35 (49%)
Fulfilled CAPS diagnostic criteria (Kuemmerle-Deschner et al., 2017)	20 (100%)	41 (100%)	10 (100%)	71 (100%)
Fulfilled Eurofever/PRINTO classification criteria (Gattorno et al., 2019)	19 (95%)	11 (27%)	5 (50%)	35 (49%)
Fulfilled diagnostic and/or classification criteria	20 (100%)	41 (100%)	10 (100%)	71 (100%)

Clinical features documented during the disease course. Elevated CRP/SAA were documented during at least one flare, irrespective of the centre at which they were measured. The CAPS diagnostic criteria require elevated CRP/SAA plus ≥2 of urticaria-like rash, cold-/stress-triggered episodes, sensorineural hearing loss, musculoskeletal symptoms (arthralgia/arthritis/myalgia), chronic aseptic meningitis and skeletal abnormalities; the Eurofever/PRINTO criteria require a confirmatory NLRP3 genotype and ≥1, or a non-confirmatory genotype and ≥2, of urticaria-like rash, red eye (conjunctivitis/episcleritis/uveitis) and sensorineural hearing loss. All individual patients fulfilled the mandatory inflammatory-marker criterion and at least two of the six clinical CAPS diagnostic criteria. P/LP, pathogenic/likely pathogenic; VUS, variant of uncertain significance. Genetic testing was performed using an NGS panel of autoinflammatory disease genes and/or genome sequencing, including analysis for low-level somatic *NLRP3* variants.

Table S2A. Distribution of ADDI total scores at the initial damage assessment and corresponding risk strata (n = 71)

ADDI total score	Mild, n	Moderate, n	Severe, n	Total, n
0	48	–	–	48
1	–	3	–	3
2	–	2	9	11
3	–	–	1	1
6	–	–	3	3
7	–	–	1	1
9	–	–	1	1
10	–	–	1	1
17	–	–	2	2
**Total**	**48**	**5**	**18**	**71**

Legend: Risk strata were assigned hierarchically:

- severe risk was assigned if ≥2 points in at least one ADDI domain were given irrespective of the total score;
- moderate, total ADDI ≥1 without any domain ≥2 points;
- mild, total ADDI 0.

Total ADDI scores ranged from 1 to 2 in the moderate and from 2 to 17 in the severe stratum.

Table S2B. Individual ADDI domain and total scores of patients with damage at the initial damage assessment or with a change in risk stratum during follow-up

Patient	Phenotype	*NLRP3* class	Variant	Repro-ductive	Renal	Develop-mental	Serosal	Neuro-logical	Auditory	Ocular	Musculo-skeletal	ADDI total	Stratum, initial	Stratum, last visit
P01	CINCA/NOMID	P/LP	H358R	0	1	3	1	6	1	1	4	17	Severe	Severe
P02	CINCA/NOMID	P/LP	M664T	0	0	3	0	6	2	2	4	17	Severe	Severe
P03	MWS	P/LP	T348M	0	0	2	0	3	2	1	2	10	Severe	Severe
P04	CINCA/NOMID	VUS	N654S	0	1	0	0	6	1	1	0	9	Severe	Severe
P05	MWS	None	–	0	1	2	0	0	1	1	2	7	Severe	Severe
P06	MWS	P/LP	E311K	1	0	3	0	0	0	0	2	6	Severe	Severe
P07	CINCA/NOMID	VUS	V198M	0	0	0	0	5	1	0	0	6	Severe	Severe
P08	CINCA/NOMID	P/LP	Y570C	0	0	0	0	5	1	0	0	6	Severe	Severe
P09	MWS	VUS	Q703K	0	0	2	0	0	1	0	0	3	Severe	Severe
P10	MWS	P/LP	A439V	0	0	0	0	0	2	0	0	2	Severe	Severe
P11	FCAS	P/LP	A439V	0	0	0	0	0	0	2	0	2	Severe	Severe
P12	MWS	P/LP	A439V	0	0	2	0	0	0	0	0	2	Severe	Severe
P13	MWS	VUS	Q703K	0	0	2	0	0	0	0	0	2	Severe	Severe
P14	MWS	VUS	Q703K	0	0	2	0	0	0	0	0	2	Severe	Severe
P15	MWS	VUS	Q703K	0	0	2	0	0	0	0	0	2	Severe	Severe
P16	MWS	VUS	Q703K	0	0	0	0	0	2	0	0	2	Severe	Severe
P17	MWS	P/LP	Y859S	0	0	0	0	0	2	0	0	2	Severe	Severe
P18	MWS	P/LP	Y859S	0	0	0	0	0	2	0	0	2	Severe	Severe
P19	MWS	P/LP	A439V	0	0	0	0	0	1	1	0	2	Moderate	Moderate
P20	MWS	VUS	Q703K	0	0	0	0	0	1	1	0	2	Moderate	Moderate
P21	MWS	P/LP	D303N	0	0	0	0	0	1	0	0	1	Moderate	Moderate
P22	MWS	P/LP	E311K	0	0	0	0	0	1	0	0	1	Moderate	Moderate
P23	MWS	VUS	Q703K	0	0	0	0	0	1	0	0	1	Moderate	Moderate
P24	MWS	P/LP	T348M	0	0	0	0	0	0	0	0	0	Mild	Moderate
P25	FCAS	VUS	V198M	0	0	0	0	0	0	0	0	0	Mild	Moderate

Domain and total scores refer to the initial damage assessment (6 months after baseline). Two patients (both mild at the initial damage assessment) progressed to the moderate stratum due to progression of sensorineural hearing loss (auditory domain, 1 point). Patients without damage and without change in stratum (n = 46) are not listed (ADDI 0, mild risk at both time points). Domain maxima: reproductive 2, renal/amyloidosis 6, developmental 3, serosal 1, neurological 6, auditory 2, ocular 3, musculoskeletal 4 (total 27).

Table S3. *NLRP3* variants, number of carriers, clinical phenotype and ADDI total score at the initial damage assessment

***NLRP3* variant**	**ACMG classification**	**Patients, n**	**Phenotype, n**	**ADDI total, median (min–max) or individual value**
A439V	Pathogenic	6	MWS 5, FCAS 1	2 (0–2)
E311K	Likely pathogenic	4	MWS 4	0.5 (0–6)
T348M	Pathogenic	2	MWS 2	5 (0–10)
Y859S	Likely pathogenic	2	MWS 2	2 (2–2)
A864D	Pathogenic	1	MWS 1	0
D303N	Pathogenic	1	MWS 1	1
H358R	Likely pathogenic	1	CINCA/NOMID 1	17
M664T	Pathogenic	1	CINCA/NOMID 1	17
P200T	Pathogenic	1	MWS 1	0
Y570C	Pathogenic	1	CINCA/NOMID 1	6
Q703K	VUS	35	MWS 31, FCAS 4	0 (0–3)
V198M	VUS	5	MWS 3, FCAS 1, CINCA/NOMID 1	0 (0–6)
N654S	VUS	1	CINCA/NOMID 1	9
No identifiable variant	–	10	FCAS 5, MWS 5	0 (0–7)

Values for variants carried by a single patient are individual scores. Owing to the small number of patients per variant, these data are descriptive.
